# Good DNA- and sufficient RNA integrity in formalin-free paraffin-embedded tissues

**DOI:** 10.1371/journal.pone.0356001

**Published:** 2026-08-14

**Authors:** Maarten Niemantsverdriet, Natalie Methorst, Sanne Yvet Hoek, Ageeth Knol, Agnes Marije Hoogland

**Affiliations:** Isala, Department of Pathology, Dr. Van Heesweg 2, AB Zwolle, GK Zwolle, The Netherlands; Sultan Qaboos University College of Medicine and Health Science, OMAN

## Abstract

In recent decades, the field of pathology has been enriched by the development of molecular biology, enabling increased specificity in cancer diagnosis and personalized therapy based on the molecular profile of tumor cells. Traditional tissue embedding techniques, routinely using toxic and even carcinogenic substances, in particular formaldehyde, are still standard practice for the field of pathology and consequently for molecular pathology. Formaldehyde damages DNA and RNA, resulting in suboptimal molecular diagnostics, possibly impacting cancer diagnosis and treatment quality. A novel tissue embedding technique based on supercritical CO_2_ can be used in a formaldehyde-free manner, producing high-quality samples suitable for basic pathology techniques. Early data indicates that the DNA isolated from tissues processed in this manner can be used for molecular pathology, but detailed DNA analysis lacked, and RNA had not been analyzed at all. Here we performed integrity analysis of both the DNA and RNA isolated from tissues 7 years, 2 years, and 3 days after embedding and compared non-fixed and formalin-fixed tissues. The results show that this novel embedding technique, used in xylene and formalin-free manner, results in DNA of higher quality than that of conventionally processed tissues, as shown by higher DIN values. On the other hand, RNA quality, as shown by RIN values, was generally lower in 3-day and 2-year old, non-fixed (NFPE) tissues compared to formalin-fixed (FFPE) tissues but similar in 7 year-old tissues. RNA isolated from 3-day, 2-year, and even most 7-year-old non-formalin fixed tissues was still suitable for the main downstream molecular technique Next-Generation-Sequencing.

## Introduction

Molecular analysis has become increasingly important for specific diagnosis, prognosis, and therapy choice for the field of pathology, especially for cancer [1]. Worldwide, tissues are routinely processed in pathology laboratories by Formalin-Fixed and Paraffin-Embedding (FFPE) with the intention to preserve tissue morphology and enable immunohistochemical analysis [2,3]. This process uses xylene, which has been shown to cause toxic effects in immunological, gastrointestinal, respiratory, developmental, reproductive, and CNS systems [4], and formaldehyde, which can induce severe health problems, including pneumonia, bronchitis, dermatitis, congenital defects, and cancer [5–7]. Increasingly important, considering the more prominent role of molecular biology in the field of pathology, is that formalin fixation also negatively impacts DNA and RNA quality. Formalin causes: DNA fragmentation, stalling of polymerases and inhibiting denaturation, and non-reproducible C > T/G > A sequencing artifacts [8–10], and RNA isolated from formalin tissue suffers from strand breakage and cross-linking [11]. These affect the quality of DNA and RNA, and thereby potentially reduce the quality of diagnosis, prognosis, and therapy choice based on molecular analysis [8,12,13].

In recent years, toxicity levels for formalin were upgraded to carcinogen grade 1B and mutagen grade 2, and formalin was banned in Europe for multiple applications, and tight restrictions were applied for almost all other uses [14–17]. A noteworthy exception to these regulations is the use of formalin in the field of pathology, because the current formalin-free tissue embedding methods do not produce pathology specimens with results of similar high quality as formalin-fixed tissues [17–19].

With the intention to eliminate the use of the toxic solvent xylene from the embedding process, a bold novel tissue processing method was developed, using supercritical Carbon dioxide (CO_2_) as an intermediate [20]. We recently showed that this method can also be used in a formalin and- xylene-free manner to produce high-quality Non-Fixed Paraffin Embedded (NFPE) samples for the basic pathology techniques, histochemistry, immunohistochemistry, and immunofluorescence [21]. Also, DNA analysis using basic molecular techniques indicates that the DNA in these NFPE tissues is of better quality than tissues processed in a conventional manner (FFPE), using formaldehyde and xylene [22]. This may indicate that it is possible to create paraffin embedded tissues that can be stained using histochemistry, immunohistochemistry, and immunofluorescence without loss of quality and perform molecular diagnostics of even higher than current standard quality without the use of toxic xylene and carcinogenic formaldehyde [21,22]. We previously reported that, in addition to these advantages, this method is cheaper and faster than processing tissues in the conventional manner [22]. However, a comparison of DNA and RNA integrity data on non-fixed, xylene-free tissues (NFPE) and Formalin-Fixed tissues (FFPE) at different timepoints after embedding were lacking, and the suitability of RNA isolated from NFPE for downstream molecular techniques had not been studied previously [22]. Evaluation of DNA and RNA integrity from paraffin-embedded tissue for assessment of suitability for downstream molecular techniques is typically done using a Tapestation 4200 (Agilent) by giving DIN values for DNA and RIN values for RNA, ranging from 10 (totally intact) and 1 (totally degraded) [23]. In the study presented here, we studied DNA and RNA integrity in formalin- and xylene-free supercritical CO_2_ processed tissue samples and assessed the suitability of NFPE RNA for the main downstream technique Next-Generation-Sequencing.

## Methods and materials

### Tissue processing

After receiving fresh tissue from the operation room at the department of Pathology, a pathologist inspected the tissue and designated the parts needed to make the diagnose, according to WHO and local protocols, and assigned the parts that could be appointed as residual tissue. Parts of the residual tissues were taken, anonymized, and used for tissue library building in this study. Available tissues were used for this study; colon, placenta, cervix, uterus, ovarium, testis, Gastrointestinal Stromal Tumor (GIST), endometrium, Colorectal Carcinoma (CRC), mamma, urothelia, adrenal, myocard, and prostate tissues were used. To reduce tissue-specific variations we excluded (spongy) tissues with known high levels of RNAse activity such as pancreas, spleen, lung, liver and small intestine [24,25]. For the 7-year-old tissues and the 3-day old tissues, one part was incubated in formalin for 24 hours and processed as formalin-fixed paraffin-embedded (FFPE) as described in Niemantsverdriet et al [21,22]. Another part was freshly processed in the TISPA I tissue processor (Tispa medical); non-fixed paraffin embedded (NFPE), as described in Niemantsverdriet et al [21,22]. For 2-year-old tissue samples, one part was incubated in formalin for 24 hours and processed in a TISPA II processor (Tispa medical), basically as described for NFPE in Niemantsverdriet et al [21,22]. Another part was freshly processed in the TISPA II tissue processor (Tispa medical); non-fixed paraffin embedded (NFPE), as described in Niemantsverdriet et al [21,22]. The TISPA II machine has a higher tissue capacity, but processing time, temperature and pressure were set identical for processing tissues with TISPA I and TISPA II. For all processed tissues, matching FFPE and NFPE blocks were stored under exactly the same conditions, in boxes, continuous and frequently monitored (manually), at room temperature and in the dark. This way, for all tissues analyzed, the same tissue specimen was split for comparative processing, and samples were paired/matched between FFPE and NFPE conditions. Because of sample size limitations, we pooled the results to get a clearer general view. Individual datapoints of matched/paired NFPE/FFPE data are shown in supplementary data Fig 1. For all series, 8–12 3µm sections were cut and basic hematoxylin and eosin (HE) staining was performed on the first and last slide for general tissue assessment.

**Fig 1:**
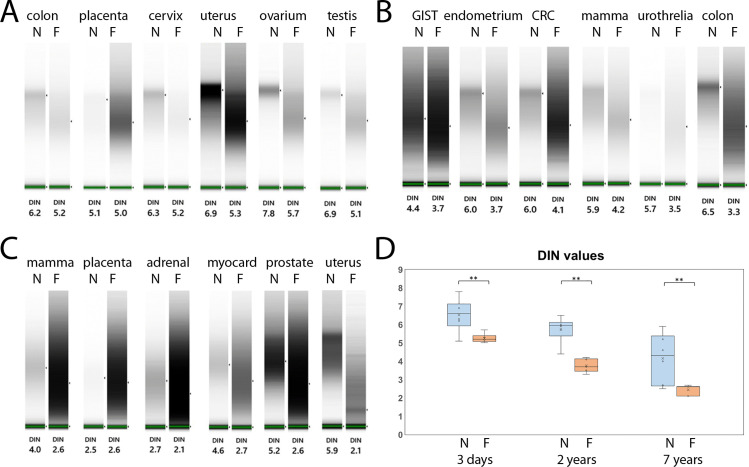
DNA integrity of non-fixed (N) and formalin-fixed (F) tissues 3 days (A), 2 years (B), and 7 years (C) after embedding. Graphical representation of DNA integrity of non-fixed (N) and formalin-fixed (F) tissues 3 days, 2 years, and 7 years after embedding. D) Boxplot representation of DIN values of formalin-fixed and non-formalin-fixed tissues. Each data point consists of 6 tissues. T-Test **: p <= 0.01 (very significant difference between groups).

### DNA and RNA isolation and analysis

DNA and RNA were isolated from 6 slides between the HE first and last, using the AllPrep DNA/RNA FFPE kit (QIAGEN). First, slides were deparaffinated using a Leica XL stainer. The pellet DNA was incubated O/N with 180 µl ATL-buffer and 40 µl proteïnase K at 56 °C, following isolation according to the QIAGEN AllPrep DNA/RNA FFPE kit standard protocol. After isolation of DNA and RNA, concentration was determined using the Qubit™ 4.0 Fluorometer. DNA or RNA fragment size and the respective DIN or RIN values were determined using the Agilent 4200 TapeStation according to the manufacturer’s instructions. Because of sample size limitations, we pooled the DIN and RIN results to get a general view. Individual RNA and DNA concentrations, RIN and DIN datapoints of matched/paired NFPE/FFPE data are shown in supplementary data S1 File.

### RNA next-generation-sequencing

Initial RNA analysis by Next-Generation-Sequencing was performed with Ion Torrent ONCOMINE FOCUS RNA panel (Thermofisher) libraries using the Oncomine Focus DNA Ampliseq 16 kit (Thermofisher) and Ion Chef System (Thermofisher) according to the manufacturer’s instructions. The library yield was quantified using the Ion Library Taqman Quantitation kit (Thermofisher) (library qPCR) according to the manufacturer’s instructions. NGS was performed on an Ion GeneStudio S5 plus (Thermofisher) according to the manufacturer’s instructions, and analysis was performed using Torrent Suite 5.12.1 software (Thermofisher). NGS data was analyzed using Ion Reporter 5.12.3.0 software (Thermofisher). RNA samples showing a positive fusion overall call in the Oncomine Focus panel were analyzed with the Archer Fusionplex lung V2 (Thermofisher) panel, using DNA Ampliseq 16 kit (Thermofisher) and Ion Chef System (Thermofisher) according to the manufacturer’s instructions. To avoid sampling variability/tumor heterogeneity, we used samples from the exact RNA extraction as used for the Focus assay. Archer data was analyzed using the Archer Analysis program (Archer), using standard settings. QC pass criteria were according to the manufacturers standard settings; TotalMappedFusionPanelReads>5000 for the ONCOMINE FOCUS RNA panel (Thermofisher), and Average Unique RNA Start Sites per Control GSP2 >= 10.0 and Number of Reads After Trimming Adapters >500.000 for the Archer Fusionplex lung V2 (Thermofisher) panel. NGS sequencing BAM files are available on request.

### Guidelines and regulations

All methods were carried out in accordance with relevant guidelines and regulations. The Daily Board of the Medical Ethics Committee Isala Zwolle (The Netherlands), has reviewed the above-mentioned research proposal with METC file nr. 180107. As a result of this review, the Committee informs you that the rules laid down in the Medical Research Involving Human Subjects Act (also known by its Dutch abbreviation WMO), do not apply to this research proposal. The need for informed consent documentation was waived and all experimental protocols were approved as part of the ethics committee approval.

## Results

Tissue was collected from colon, placenta, cervix, uterus, ovarium, testis, GIST, endometrium, CRC, mamma, urothelia, adrenal, myocard, and prostate, based on availability. Tissues were split, and one part was fixed in formalin for 24 hours. Tissues were either Formalin-Fixed and Paraffin-Embedded (FFPE) using a conventional processing technique (groups 1 and 3) or a supercritical CO_2_-based technique (group 2). The matching tissues for all three groups were processed xylene-free and unfixed as Non-Fixed Paraffin-Embedded tissue (NFPE) using a supercritical CO_2_ based technique.

### DNA and RNA integrity

**Fig 2:**
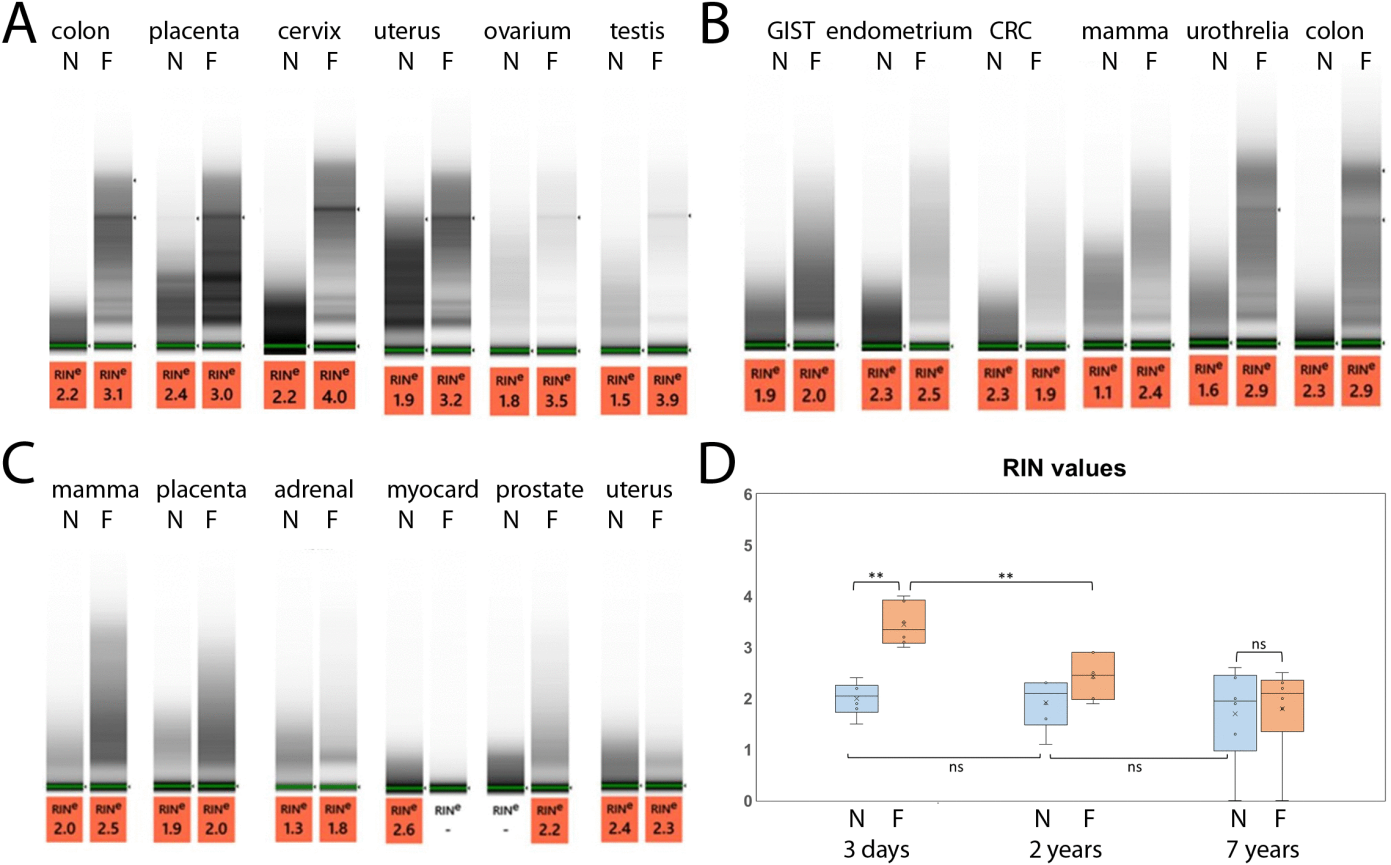
RNA integrity of non-fixed (N) and formalin-fixed (F) tissues 3 days (A), 2 years (B) and 7 years (C) after embedding. Graphical representation of DNA integrity of non-fixed (N) and formalin-fixed (F) tissues 3 days, 2 years, and 7 years after embedding. D) Boxplot representation of RIN values of formalin-fixed and non-formalin-fixed tissues. Each data point consists of 6 tissues. T-Test **: p <= 0.01 (very significant difference between groups), ns: p > 0.5 (no significant difference between groups).

7 years (group 1), 2 years (group 2), or 3 days (group 3) after embedding, DNA (Fig 1) and RNA (Fig 2) were isolated, and the integrity was analyzed using a Tapestation 4200 (Agilent) device.

### RNA NGS

RNA functionality of 3–days-old, 2-year-old, and 7-year-old FFPE (formalin-fixed) and NFPE (non-fixed) samples was analyzed using the Thermofisher focus RNA panel, a commercial NGS panel, designed to detect targeted fusions and exon skipping events in 23 genes and additionally imbalance assays for four of these genes, ALK, ROS1, RET, and NTRK1, to detect non-targeted fusions. In the primary RNA analysis, 3 of 18 FFPE samples did not pass the QC criteria (Table 1).

**Table 1:** RNA NGS analysis of Non-fixed and Formalin-Fixed tissues.

3 days						
*Tissue*	*Formalin-fixed*	*Fusion Sample QC*	*Fusion Overall Call*	*Additional test*	*result*	*Conclusion RNA NGS*
Colon	NO	PASS	NEGATIVE	none		OK
	YES	PASS	NEGATIVE	none		OK
Placenta	NO	PASS	NEGATIVE	none		OK
	YES	PASS	NEGATIVE	none		OK
Cervix	NO	PASS	NEGATIVE	none		OK
	YES	PASS	**POSITIVE [DriverGene = MET(13) – MET(15)] 254**	Archer	NEGATIVE	**Discordant**
Uterus	NO	PASS	NEGATIVE	none		OK
	YES	PASS	NEGATIVE	none		OK
Ovarium	NO	PASS	NEGATIVE	none		OK
	YES	PASS	**POSITIVE [DriverGene = EGFR(1) – EGFR(8)] 500**	Archer	NEGATIVE	**Discordant**
Testis	NO	PASS	NEGATIVE	none		OK
	YES	PASS	NEGATIVE	none		OK
**2 years**						
*Tissue*	*Formalin-fixed*	*Fusion Sample QC*	*Fusion Overall Call*	*Additional test*	*result*	*Conclusion RNA NGS*
GIST	NO	PASS	NEGATIVE	none		OK
	YES	PASS	NEGATIVE	none		OK
Endometrium	NO	PASS	NEGATIVE	none		OK
	YES	PASS	NEGATIVE	none		OK
CRC	NO	PASS	NEGATIVE	none		OK
	YES	PASS	**POSITIVE [DriverGene = ALK,Imbalance] 0,346**	Archer	NEGATIVE	**Discordant**
Mamma	NO	PASS	NEGATIVE	none		OK
	YES	PASS	NEGATIVE	none		OK
Urothelia	NO	PASS	NEGATIVE	none		OK
	YES	PASS	**POSITIVE [DriverGene = MET(13) – MET(15)] 991**	Archer	NEGATIVE	**Discordant**
Colon	NO	PASS	NEGATIVE	none		OK
	YES	PASS	NEGATIVE	none		OK
**7 years**						
*Tissue*	*Formalin-fixed*	*Fusion Sample QC*	*Fusion Overall Call*	*Additional test*	*result*	*Conclusion RNA NGS*
Mamma	NO	PASS	NEGATIVE	none		OK
	YES	PASS	**POSITIVE [DriverGene = RET, Imbalance] 2822,4044**	Archer	NEGATIVE	**Discordant**
Placenta	NO	PASS	NEGATIVE	none		OK
	YES	PASS	NEGATIVE	none		OK
Prostate	NO	**FAIL**	**FAIL**	none		**FAIL**
	YES	**FAIL**	**FAIL**	none		**FAIL**
Myocard	NO	PASS	NEGATIVE	none		OK
	YES	**FAIL**	**FAIL**	none		**FAIL**
Uterus	NO	PASS	NEGATIVE	none		OK
	YES	**FAIL**	**FAIL**	none		**FAIL**
Adrenal	NO	PASS	**POSITIVE [DriverGene = ALK,Imbalance] 0,163**	Archer	NEGATIVE	**Discordant**
	YES	PASS	NEGATIVE	none		OK
				**Failures**	Formalin-Fixed	3
					Non-Fixed	1
				**Discordant**	Formalin-Fixed	5
					Non-Fixed	1

For NFPE, only the prostate sample, tissue that also did not pass for FFPE, did not pass the QC requirements (Table 1). All failed samples were in the 7-year-old series. 5 of 18 FFPE samples showed a positive signal in the fusion overall call versus only 1 of 18 NFPE samples. The fusions implied in FFPE were imbalances in ALK and RET, two times MET exon 14 skipping, and an EGFR(1)-EGFR(8) fusion. The only positive result in NFPE was an ALK imbalance in the 7-year-old adrenal tissue. Since in all 6 cases the tissue counterpart, processed with the alternative method was negative for this fusion, and the suggested fusions, combined with the relatively low read counts (S1 File), were previously identified in a diagnostic setting as potentially false-positive, we consequently tested the RNA with the Archer fusionplex lung V2 panel. In all 6 cases, the alternative test was negative (Table 1 and S2 File).

## Discussion

Most new cancer drugs target dysregulated genes that promote hallmarks of cancer, such as tumor initiation, proliferation and cancer progression [26]. These drugs, designated ‘targeted therapies’, are prescribed based on highly specific mutations or alterations detected in the DNA or RNA of the tumor. They specifically alter the function of a molecular target with a role in cancer pathogenesis [26]. Since these targeted therapies are designed to counteract the effects of very specific DNA and/or RNA alterations, variations in as little as a single basepair in a genome of approximately 3 billion (3.000.000.000) basepairs, it is imperative that the DNA and RNA isolated from tumor cells is free of tissue-processing-induced damage to the DNA or RNA that may alter the conclusion of the analysis [10].

Formaldehyde (formalin) fixation is a standard procedure and is routinely performed in pathology laboratories all over the world. It has been demonstrated that DNA is damaged by formalin fixation, causing fragmentation, less efficient amplification by polymerases, non-reproducible sequencing artifacts, and that those factors can be formed at varying proportions among different laboratories [6,12]. In addition to damage by formaldehyde in DNA, it has been shown that the RNA isolated from formalin fixed tissues shows damage analogous to DNA damage [11].

We used a novel technique, non-formalin processed paraffin-embedded tissues (NFPE), to process tissues without formalin fixation and compared the integrity of the DNA and RNA to matching tissues that have been processed with formalin fixation. DNA and RNA were isolated 7 years, 2 years, or three days after embedding. Three days after embedding DNA processed as NFPE had a significantly higher integrity than the FFPE processed counterpart (Fig 1). The integrity of DNA declined after two years and even further after 7 years, but in all three timepoints, the integrity of NFPE was significantly higher than the FFPE counterpart (Fig 1). The DNA integrities for our FFPE samples are in line with (or possibly even a little higher) than integrities published previously by others [27,28] for FFPE material, showing that not lower than usual FFPE integrity in our samples, but higher NFPE integrity is the cause of this difference. This is in line with the data we previously published, that the DNA of older NFPE processed tissues, 5 years-old at the time, is still very suitable for Next-Generation-Sequencing, shows less degradation than DNA isolated from FFPE counterparts, has a higher relative DNA yield, better NGS library prep, and are less likely to acquire formalin fixation sequencing artifacts [22]. In the current study, we show that, in contrast to DNA, RNA integrity in NFPE material is lower than that of FFPE in freshly embedded and 2-year old tissues, but integrity appears to decrease more slowly in time than RNA isolated from FFPE as 7-year old samples show similar RNA integrity for NFPE and FFPE (Fig 2). Even though RNA integrity numbers were lower for NFPE, RNA Next-Generation-Sequencing using a small commercial panel was generally successful, even in most older samples and in this cohort even more successful than RNA NGS on FFPE material (Table 1). 5 of 18 FFPE samples (28%) and 1 of 18 NFPE (6%) samples showed a positive fusion call in the initial RNA analysis. These positive fusion results found in FFPE material were in ALK, RET, MET, and EGFR (Table 1 and S1 File), all Tyrosine kinases. The one positive result in NFPE was an ALK imbalance in the 7-year-old adrenal tissue (Table 1 and S1 File). Since in all cases the tissue part processed with the alternative method was negative for this fusion, a false positive result was considered a possibility, and we tested the RNA with the Archer FusionPlex assay. This assay uses the anchored multiplex PCR (AMP) chemistry, which is highly sensitive and specific; it can very reliably detect low-abundance transcripts and in the case of true-positive fusions are unlikely to give a negative result [29,30]. In all 6 cases, the alternative test was negative, showing discrepancy between the two tests (Table 1 and S1 File).

Tyrosine kinases including ALK, RET, MET, and EGFR, regulate key cellular processes, including cell proliferation, differentiation and survival, and when altered by specific mutations, can all be targeted with alteration specific Tyrosine Kinase Inhibitors (TKI) [31]. These TKI are very expensive, cost from a few thousand dollars a month to several hundred thousand dollars a year [32] and only work efficiently if the specific targeted DNA or RNA alterations are present but are not at all effective if these alterations are not present [31,32].

In addition to the effect on RNA presented in the current study, our previous work also indicates that NFPE may be less prone to acquire formalin sequencing artefacts in DNA [22] that potentially falsely identify oncogenic mutations in tyrosine kinases [8]. Therefore, reducing the number of false-positive results for oncogenic tyrosine kinase by using NFPE instead of formalin-fixed tissues would improve the quality of personalized therapy based on both RNA and DNA Next-Generation-Sequencing.

To our knowledge, our laboratory is the first to study DNA and RNA of tissues embedded without using xylene and formalin fixation by using the NFPE method based on supercritical CO_2_. So far, our previous results [21,22] and the results presented here show that processing tissues as NFPE instead of FFPE has great potential. However, the number of samples available from our initial studies is limited, and unfortunately, only a small number of suitable samples were available for the current study. To get a general view of the behavior of RNA and DNA in NFPE and FFPE tissues at different timepoints, we pooled different tissues. Although there is an intrinsic variation between tissue types in DNA and RNA integrity that may cause some variation, individual comparisons of RIN and DIN values between the tissues split for NFPE and FFPE processing show the same trend as the main figures (supplementary data S1 File). Broader validation of the NFPE tissues, and the effects on DNA and RNA across multiple other pathology laboratories would be very important to verify and substantiate our claims. To achieve larger accessibility to this method, it would be necessary to commercially produce machines capable of embedding tissues using supercritical CO_2_ on a larger scale, and a technical support team that can achieve a level of support comparable to the level for embedding machines used in a diagnostic pathology setting.

## Conclusion

Our results show that xylene- and formalin-free processing of tissues (NFPE) is possible and produces DNA with higher integrity than DNA of formalin-fixed tissues. RNA integrity in NFPE was lower than RNA integrity of FFPE in 3-days and 2-year old samples, but similar in 7 year old samples. RNA Next-Generation-Sequencing was successful using NFPE RNA, even in most 7-year-old samples, and may be less prone to sequencing artifacts than RNA isolated from tissue processed after formalin fixation. This suggests that processing tissue as NFPE blocks, which is quicker and cheaper than traditional Formalin-Fixed Paraffin-Embedding of tissues [22], has the potential to drastically decrease the use of toxic xylene and, moreover, carcinogenic formaldehyde in pathology labs throughout the world and may even simultaneously improve molecular pathology. Since the number of samples available from our initial studies is limited and we are currently the only laboratory testing these NFPE tissues, further multicenter validation, broader tissue-type testing, and workflow implementation studies may be needed before clinical adoption.

## Supporting information

S1 File NFPE/FFPE paired values and analysis. (PDF)

S2 File Additional RNA analysis. (PDF)
