# Supplementary material for: Good DNA- and sufficient RNA integrity in formalin-free paraffin-embedded tissues
Source: PLoS One. 2026 Aug 14;21(8):e0356001. doi: 10.1371/journal.pone.0356001 (PMC13475849; doi:10.1371/journal.pone.0356001)
Supplement: Supplementary file 1 [file pone.0356001.s001.pdf]

| 3 days | | | | | | | | |
|---|---|---|---|---|---|---|---|---|
| Method | Tissue | tumour/normal | Cold-ischemia time (hrs) | Formalin fixation time (hrs) | RNA(ng/µl)* | DNA(ng/µl)* | DIN | RIN |
| NFPE | Colon | Normal | 60 | | 276 | 157 | 6,2 | 2,2 |
| FFPE | Colon | Normal | 60 | 24 | 198,4 | 457 | 5,2 | 3,1 |
| | | | | | | | | |
| NFPE | Placenta | Normal | 2 | | 93,2 | 47 | 5,1 | 2,4 |
| FFPE | Placenta | Normal | 2 | 46 | 93,8 | 230 | 5 | 3 |
| | | | | | | | | |
| NFPE | Cervix | Normal | 4,25 | | 620 | 1400 | 6,3 | 2,2 |
| FFPE | Cervix | Normal | 4,25 | 48 | 7,88 | 327 | 5,2 | 4 |
| | | | | | | | | |
| NFPE | Uterus | Normal | 3 | | 64 | 682 | 6,9 | 1,9 |
| FFPE | Uterus | Normal | 3 | 3 | 250 | 132 | 5,3 | 3,2 |
| | | | | | | | | |
| NFPE | Ovarium | Normal | 17,5 | | 446 | 378 | 7,8 | 1,8 |
| FFPE | Ovarium | Normal | 17,5 | 24 | 418 | 418 | 5,7 | 3,5 |
| | | | | | | | | |
| NFPE | Testis | Normal | 65 | | 96,2 | 49,2 | 6,9 | 1,5 |
| FFPE | Testis | Normal | 65 | 22 | 408 | 430 | 5,1 | 3,9 |

| 2 Year | | | | | | | | |
|---|---|---|---|---|---|---|---|---|
| Method | Tissue | | | | RNA(ng/µl)* | DNA(ng/µl)* | DIN | RIN |
| NFPE | GIST | Tumour | 17 | | 385 | 77 | 4,4 | 1,9 |
| FFPE | GIST | Tumour | 17 | 72 | 510 | 228 | 3,7 | 2 |
| | | | | | | | | |
| NFPE | endometrium | Tumour | 2,5 | | 540 | 18,7 | 6 | 2,3 |
| FFPE | endometrium | Tumour | 2,5 | 23 | 860 | 302 | 3,7 | 2,5 |
| | | | | | | | | |
| NFPE | colon | Tumour | 2,5 | | 410 | 17,5 | 6 | 2,3 |
| FFPE | colon | Tumour | 2,5 | 46 | 930 | 143 | 4,1 | 1,9 |
| | | | | | | | | |
| NFPE | mamma | Tumour | 3,25 | | 114 | 20,9 | 5,9 | 1,1 |
| FFPE | mamma | Tumour | 3,25 | 22 | 79,5 | 19,7 | 4,2 | 2,4 |
| | | | | | | | | |
| NFPE | urothelia | Tumour | 0,25 | | 172,2 | 17,4 | 5,7 | 1,6 |
| FFPE | urothelia | Tumour | 0,25 | 27,5 | 860 | 331 | 3,5 | 2,9 |
| | | | | | | | | |
| NFPE | colon | Tumour | 2,5 | | 250 | 22,7 | 6,5 | 2,3 |
| FFPE | colon | Tumour | 2,5 | 48 | 330 | 54,9 | 3,3 | 2,9 |

| 7 year | | | | | | | | |
|---|---|---|---|---|---|---|---|---|
| Method | Tissue | | | | RNA(ng/µl)* | DNA(ng/µl)* | DIN | RIN |
| NFPE | Mamma | Tumour | 94 | | 101 | 89,8 | 4 | 2 |
| FFPE | Mamma | Tumour | 94 | 24 | 296 | 67,7 | 2,6 | 2,5 |
| | | | | | | | | |
| NFPE | Placenta | Normal | 2 | | 79,9 | 1,95 | 2,5 | 1,9 |
| FFPE | Placenta | Normal | 2 | 48 | 283 | 77,9 | 2,6 | 2 |
| | | | | | | | | |
| NFPE | Prostate | Normal | 25 | | 302 | 98,7 | 5,2 | 0,1 |
| FFPE | Prostate | Normal | 25 | 22 | 194 | 75,6 | 2,6 | 2,2 |
| | | | | | | | | |
| NFPE | Myocard | Normal | 25 | | 247 | 11,4 | 4,6 | 2,6 |
| FFPE | Myocard | Normal | 25 | 22 | 82,9 | 28,8 | 2,7 | 0,1 |
| | | | | | | | | |
| NFPE | Uterus | Normal | 1,5 | | 368 | 51,6 | 5,9 | 2,4 |
| FFPE | Uterus | Normal | 1,5 | 23 | 61,7 | 59,5 | 2,1 | 2,3 |
| | | | | | | | | |
| NFPE | Adrenal | Normal | 94 | | 46,1 | 1,39 | 2,7 | 1,3 |
| FFPE | Adrenal | Normal | 94 | 23 | 24 | 8,76 | 2,1 | 1,8 |

* DNA and RNA were isolated using a column based-method suitable for tapestion measurements but where concentrations are not typically correlated with tissue surface

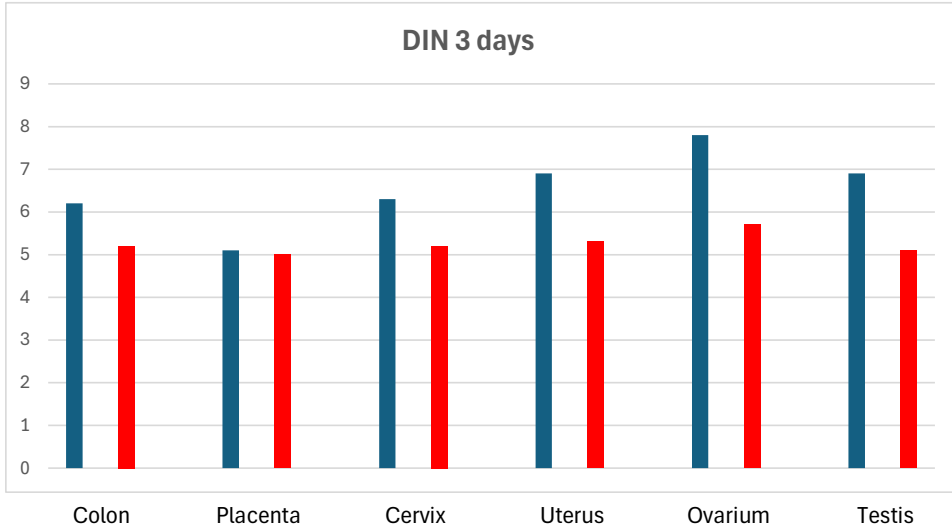


FFPE-blue NFPE-Red

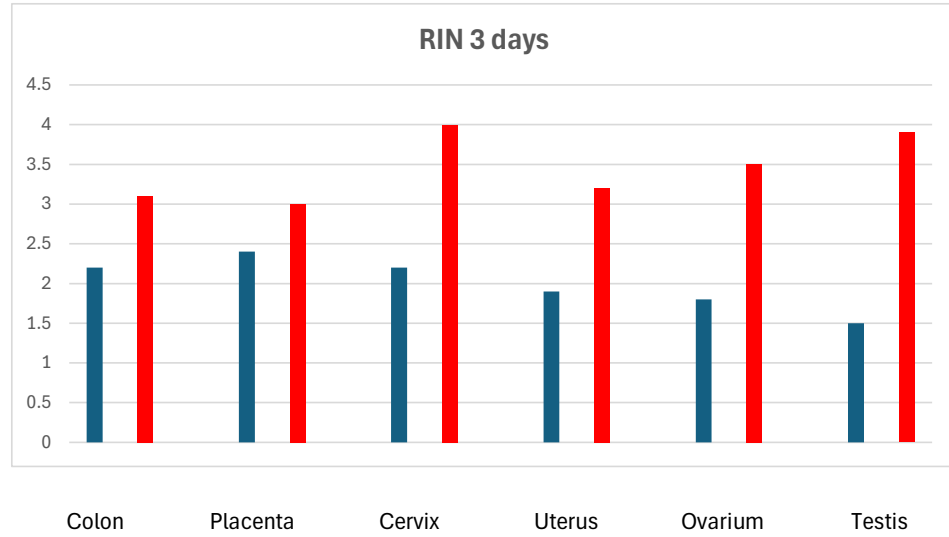


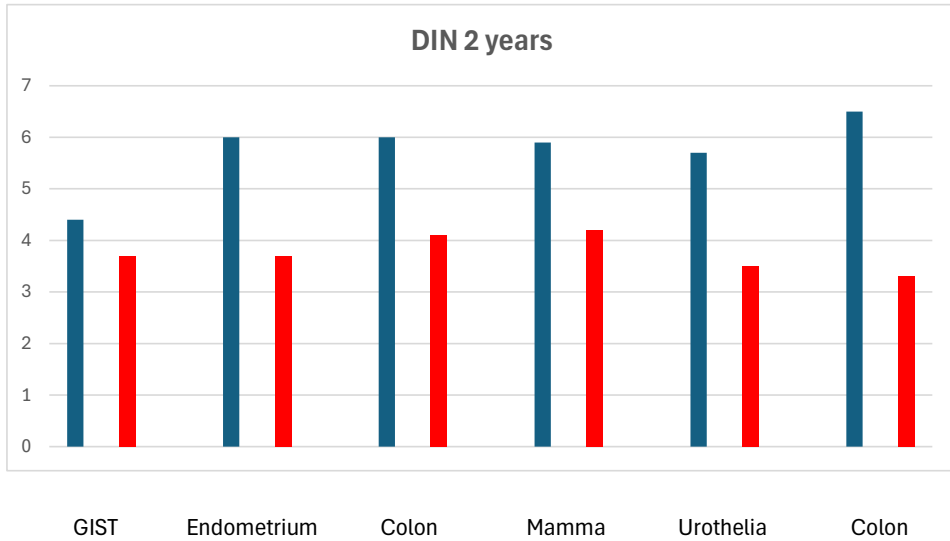


FFPE-blue NFPE-Red

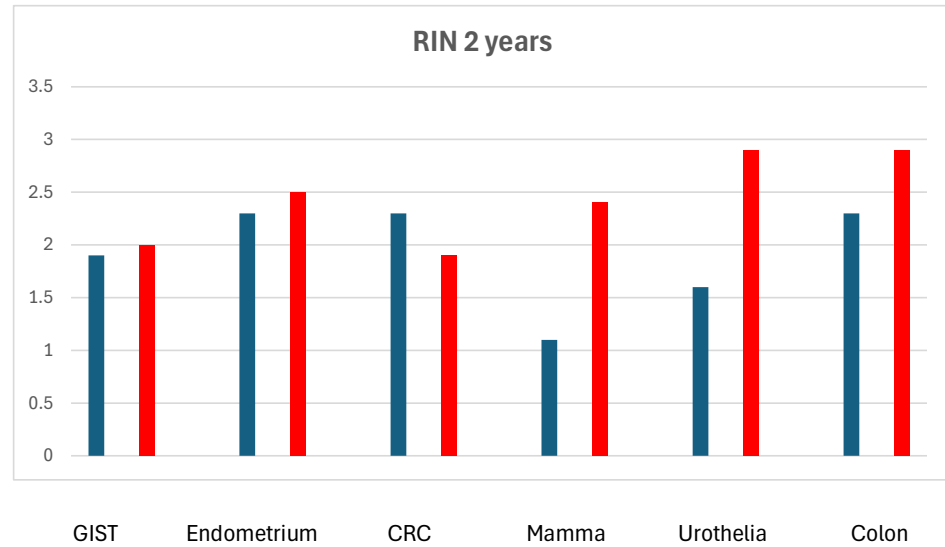


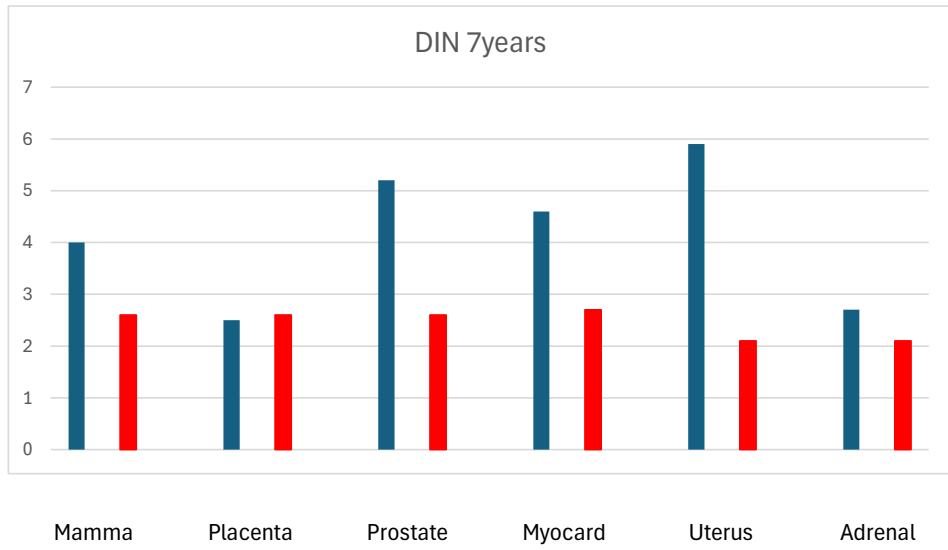


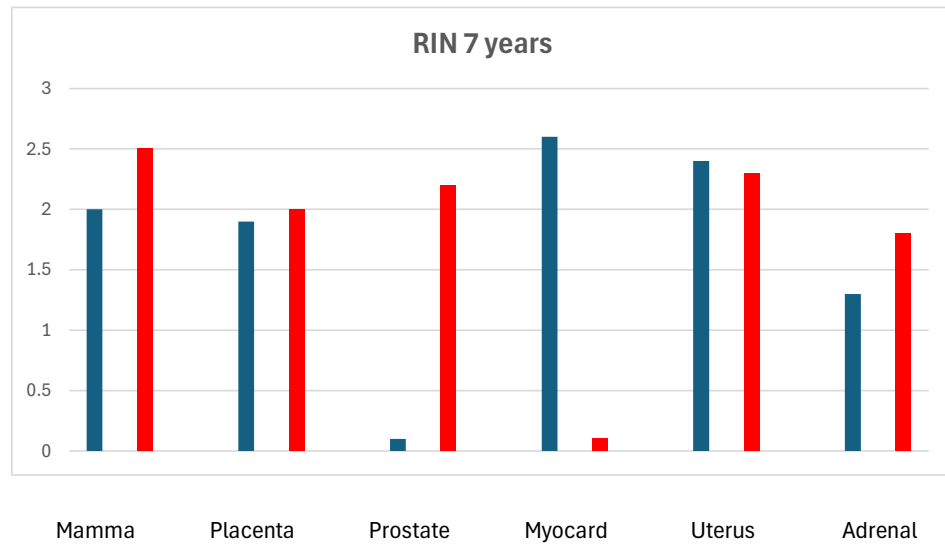


FFPE-blue NFPE-Red
