# Supplementary material for: Good DNA- and sufficient RNA integrity in formalin-free paraffin-embedded tissues
Source: PLoS One. 2026 Aug 14;21(8):e0356001. doi: 10.1371/journal.pone.0356001 (PMC13475849; doi:10.1371/journal.pone.0356001)
Supplement: Supplementary file 2 [file pone.0356001.s002.pdf]

3 days

Focus RNA analysis

Cervix Formalin-Fixed

Analysis Results

MyVariants | Download | Visualize | Selected Variants | Send to Report Role | Switch To | Generate Report

Analysis Name: [redacted] **Cancer Type**: Unknown **Sample ID**: N/A **Fusion Sample QC**: PASS,[TotalMappedFusionPanelReads>5000;Me… **Fusion Overall Call**: POSITIVE ,[DriverGene=MET,IsoformsDetected=…

Total Mapped Fusion Panel Reads: 213941 **Total Unmapped Reads**: 58927

To learn more about reviewing your results, visit the help guide.

Fusions

Search Go Preferences

Classification	Locus	Type	Filter	No Call Reason	Genes (Exons)	Read Counts	Oncomine Variant Class	Onc
Unclassified	chr7:116411708 - chr7:116414935	RNAExonVar	PASS		MET(13) - MET(15)	254	RNAExonVariant	Gain

Filter Options

Variants

- **Filtered In Variants (1)**
- Hidden Variants (0)
- Filtered Out Variants (297)

Ovarium Formalin-Fixed

Analysis Results

MyVariants | Download | Visualize | Selected Variants | Send to Report Role | Switch To | Generate Report

Analysis Name: [redacted]-12-… **Cancer Type**: Unknown **Sample ID**: N/A **Fusion Sample QC**: PASS,[TotalMappedFusionPanelReads>5000;Me… **Fusion Overall Call**: POSITIVE ,[DriverGene=EGFR,IsoformsDetected…

Total Mapped Fusion Panel Reads: 334992 **Total Unmapped Reads**: 92213

To learn more about reviewing your results, visit the help guide.

Fusions

Search Go Preferences

Classification	Locus	Type	Filter	No Call Reason	Genes (Exons)	Read Counts	Oncomine Variant Class	Onc
Unclassified	chr7:55087058 - chr7:55223523	RNAExonVar	PASS		EGFR(1) - EGFR(8)	500	RNAExonVariant	Gain

1 | 15 items per page | 1 - 1 of 1 items

Filter Options

Variants

- **Filtered In Variants (1)**
- Hidden Variants (0)
- Filtered Out Variants (297)

Samples

- **Fusions Sample**: [redacted]
  - **Cancer Type** : Unknown
  - **Gender** : Unknown
  - **Percentage Cellularity** : 100
  - **Sample Type** : RNA

2 years

Focus RNA analysis

CRC Formalin-Fixed 2 year

Analysis Results

MyVariants | Download | Visualize | Selected Variants | Send to Report Role | Switch To | Generate Report

Analysis Name: [redacted] **Cancer Type**: Unknown **Sample ID**: N/A **Fusion Sample QC**: PASS,[TotalMappedFusionPanelReads>5000;Me... **Fusion Overall Call**: POSITIVE ,[DriverGene=ALK,IsoformsDetected=... **Total Mapped Fusion Panel Reads**: 150058 **Total Unmapped Reads**: 126925

To learn more about reviewing your results, visit the help guide.

Fusions

Search | Go | Preferences

Classification	Locus	Type	Filter	No Call Reason	Genes (Exons)	Read Counts	Oncomine Variant Class	Oncomine Gene Class	De
Unclassified	chr10:43606730, chr10:43622086	ASSAYS_5P	FAIL		RET	1693,0			LC
Unclassified	chr1:156834532, chr1:156851323	ASSAYS_5P	FAIL		NTRK1	0,1701			LC
Unclassified	chr2:29551347, chr2:29430138	ASSAYS_5P	PASS		ALK	0,346			
Unclassified	chr6:117711009, chr6:117632280	ASSAYS_5P	FAIL		ROS1	0,0			LC

Filter Options

Variants

- Filtered In Variants (0)
- Hidden Variants (0)
- Filtered Out Variants (298)

Samples

- Fusions Sample: [redacted]
  - Cancer Type : Unknown
  - Gender : Unknown
  - Percentage Cellularity : 100
  - Sample Type : RNA

Urothelia Formalin-Fixed 2 year

Analysis Results

MyVariants | Download | Visualize | Selected Variants | Send to Report Role | Switch To | Generate Report

Analysis Name: [redacted] **Cancer Type**: Unknown **Sample ID**: N/A **Fusion Sample QC**: PASS,[TotalMappedFusionPanelReads>5000;Me... **Fusion Overall Call**: POSITIVE ,[DriverGene=MET,IsoformsDetected=... **Total Mapped Fusion Panel Reads**: 245006 **Total Unmapped Reads**: 37022

To learn more about reviewing your results, visit the help guide.

Fusions

Search | Go | Preferences

Classification	Locus	Type	Filter	No Call Reason	Genes (Exons)	Read Counts	Oncomine Variant Class	Oncomine Gene Class	De
Unclassified	chr7:116411708 - chr7:116414935	RNAExonVai	PASS		MET(13) - MET(15)	991	RNAExonVariant	Gain-of-Function	

1 | 15 items per page | 1 - 1 of 1 items

Filter Options

Variants

- Filtered In Variants (1)
- Hidden Variants (0)
- Filtered Out Variants (297)

Samples

- Fusions Sample: [redacted]
  - Cancer Type : Unknown
  - Gender : Unknown
  - Percentage Cellularity : 100
  - Sample Type : RNA

7 years

Focus RNA analysis

Mamma Formalin-Fixed

Analysis Results

MyVariants | Download | Visualize | Selected Variants | Send to Report Role | Switch To | Generate Report

Analysis Name: [redacted] **Cancer Type:** Unknown **Sample ID:** N/A **Fusion Sample QC:** PASS,[TotalMappedFusionPanelReads>5000;Me... **Fusion Overall Call:** POSITIVE ,[DriverGene=RET,IsoformsDetected=... **Total Mapped Fusion Panel Reads:** 11498 **Total Unmapped Reads:** 60909

To learn more about reviewing your results, visit the help guide.

Fusions

Search Go Preferences

Classification	Locus	Type	Filter	No Call Reason	Genes (Exons)	Read Counts	Oncomine Variant Class	Oncomine Gene Class
Unclassified	chr10:43606730, chr10:43622086	ASSAYS_5P	PASS		RET	2822,4044		
Unclassified	chr1:156834532, chr1:156851323	ASSAYS_5P	FAIL		NTRK1	0,0		
Unclassified	chr2:29551347, chr2:29430138	ASSAYS_5P	FAIL		ALK	0,0		
Unclassified	chr6:117711009, chr6:117632280	ASSAYS_5P	FAIL		ROS1	0,0		

Filter Options

Variants

- Filtered In Variants (0)
- Hidden Variants (0)
- **Filtered Out Variants (298)**

Samples

- **Fusions Sample:** [redacted]
  - **Cancer Type :** Unknown
  - **Gender :** Unknown
  - **Percentage Cellularity :** 100
  - **Sample Type :** RNA

Adrenal NON-Fixed

Analysis Results

MyVariants | Download | Visualize | Selected Variants | Send to Report Role | Switch To | Generate Report

Analysis Name: [redacted] **Cancer Type:** Unknown **Sample ID:** N/A **Fusion Sample QC:** PASS,[TotalMappedFusionPanelReads>5000;Me... **Fusion Overall Call:** POSITIVE ,[DriverGene=ALK,IsoformsDetected=... **Total Mapped Fusion Panel Reads:** 86028 **Total Unmapped Reads:** 97553

To learn more about reviewing your results, visit the help guide.

Fusions

Search Go Preferences

Classification	Locus	Type	Filter	No Call Reason	Genes (Exons)	Read Counts	Oncomine Variant Class	Onc
Unclassified	chr10:43606730, chr10:43622086	ASSAYS_5P	FAIL		RET	597,1240		
Unclassified	chr1:156834532, chr1:156851323	ASSAYS_5P	FAIL		NTRK1	0,0		
Unclassified	chr2:29551347, chr2:29430138	ASSAYS_5P	PASS		ALK	0,163		
Unclassified	chr6:117711009, chr6:117632280	ASSAYS_5P	FAIL		ROS1	0,1		

Filter Options

Variants

- Filtered In Variants (0)
- Hidden Variants (0)
- **Filtered Out Variants (298)**

Samples

- **Fusions Sample:** [redacted]
  - **Cancer Type :** Unknown
  - **Gender :** Unknown
  - **Percentage Cellularity :** 100
  - **Sample Type :** RNA

Archer analysis (Fusionplex lung V2)

Sample Name	Assay Results	Fusion Results	QC Results
Adrenal_NFPE_7years<br>Low Med High<br>Unassigned<br>Review Complete	0 Fusions	Strong Fusions & Oncogenic Isoforms<br>No events found.	Sample QC<br>Fusion QC
CRC_FFPE_2years<br>Low Med High<br>Unassigned<br>Review Complete	0 Fusions	Strong Fusions & Oncogenic Isoforms<br>No events found.	Sample QC<br>Fusion QC
Cervix_FFPE_3days<br>Low Med High<br>Unassigned<br>Review Complete	0 Fusions	Strong Fusions & Oncogenic Isoforms<br>No events found.	Sample QC<br>Fusion QC
Mamma_FFPE_7years<br>Low Med High<br>Unassigned<br>Review Complete	0 Fusions	Strong Fusions & Oncogenic Isoforms<br>No events found.	Sample QC<br>Fusion QC
Ovarium_FFPE_3days<br>Low Med High<br>Unassigned<br>Review Complete	0 Fusions	Strong Fusions & Oncogenic Isoforms<br>No events found.	Sample QC<br>Fusion QC
Urothelia_FFPE_2years<br>Low Med High<br>Unassigned<br>Review Complete	0 Fusions	Strong Fusions & Oncogenic Isoforms<br>No events found.	Sample QC<br>Fusion QC
