# Supplementary material for: Good DNA- and sufficient RNA integrity in formalin-free paraffin-embedded tissues
Source: PLoS One. 2026 Aug 14;21(8):e0356001. doi: 10.1371/journal.pone.0356001 (PMC13475849; doi:10.1371/journal.pone.0356001)
Supplement: Peer review history

# Peer review: Decision Letter 0

**DOI:** 10.1371/journal.pone.0356001.r001

**Authors:** Nasar Alwahaibi

26 Apr 2026

PONE-D-26-13956

Superior DNA- and sufficient RNA integrity in formalin-free paraffin-embedded tissues

PLOS One

Dear Dr. Hoogland,

Thank you for submitting your manuscript to PLOS ONE. After careful consideration, we feel that it has merit but does not fully meet PLOS ONE’s publication criteria as it currently stands. Therefore, we invite you to submit a revised version of the manuscript that addresses the points raised during the review process.

Please submit your revised manuscript by Jun 10 2026 11:59PM. If you will need more time than this to complete your revisions, please reply to this message or contact the journal office at plosone@plos.org. When you're ready to submit your revision, log on to https://www.editorialmanager.com/pone/ and select the 'Submissions Needing Revision' folder to locate your manuscript file.

Please include the following items when submitting your revised manuscript:

- A letter that responds to each point raised by the academic editor and reviewer(s). You should upload this letter as a separate file labeled 'Response to Reviewers'.
- A marked-up copy of your manuscript that highlights changes made to the original version. You should upload this as a separate file labeled 'Revised Manuscript with Track Changes'.
- An unmarked version of your revised paper without tracked changes. You should upload this as a separate file labeled 'Manuscript'.

If you would like to make changes to your financial disclosure, please include your updated statement in your cover letter. Guidelines for resubmitting your figure files are available below the reviewer comments at the end of this letter.

If applicable, we recommend that you deposit your laboratory protocols in protocols.io to enhance the reproducibility of your results. Protocols.io assigns your protocol its own identifier (DOI) so that it can be cited independently in the future. For instructions see: https://journals.plos.org/plosone/s/submission-guidelines#loc-laboratory-protocols. Additionally, PLOS ONE offers an option for publishing peer-reviewed Lab Protocol articles, which describe protocols hosted on protocols.io. Read more information on sharing protocols at https://plos.org/protocols?utm_medium=editorial-email&utm_source=authorletters&utm_campaign=protocols.

As the corresponding author, your ORCID iD is verified in the submission system and will appear in the published article. PLOS supports the use of ORCID, and we encourage all coauthors to register for an ORCID iD and use it as well. Please encourage your coauthors to verify their ORCID iD within the submission system before final acceptance, as unverified ORCID iDs will not appear in the published article. *Only*  the individual author can complete the verification step; PLOS staff *cannot*  verify ORCID iDs on behalf of authors.

We look forward to receiving your revised manuscript.

Kind regards,

Nasar Alwahaibi, PhD

Academic Editor

PLOS One

**Journal Requirements:**

When submitting your revision, we need you to address these additional requirements.

1. Please ensure that your manuscript meets PLOS ONE's style requirements, including those for file naming. The PLOS ONE style templates can be found at

https://journals.plos.org/plosone/s/file?id=wjVg/PLOSOne_formatting_sample_main_body.pdf and

https://journals.plos.org/plosone/s/file?id=ba62/PLOSOne_formatting_sample_title_authors_affiliations.pdf

2. We note that the grant information you provided in the ‘Funding Information’ and ‘Financial Disclosure’ sections do not match.

When you resubmit, please ensure that you provide the correct grant numbers for the awards you received for your study in the ‘Funding Information’ section.

3. Thank you for stating the following in the Financial Disclosure  section:

“Initials of the authors who received each award: AMH

Grant numbers awarded to each author: 1

The full name of each funder: Novartis Nederland

URL of each funder website: https://www.novartis.com/nl-nl/

Did the sponsors or funders play any role in the study design, data collection and analysis, decision to publish, or preparation of the manuscript? No, the funders had no role in study design, data collection and analysis, decision to publish, or preparation of the manuscript.”

We note that you received funding from a commercial source: Novartis Nederland

Please provide an amended Competing Interests Statement that explicitly states this commercial funder, along with any other relevant declarations relating to employment, consultancy, patents, products in development, marketed products, etc.

Within this Competing Interests Statement, please confirm that this does not alter your adherence to all PLOS ONE policies on sharing data and materials by including the following statement: "This does not alter our adherence to PLOS ONE policies on sharing data and materials.” (as detailed online in our guide for authors http://journals.plos.org/plosone/s/competing-interests).  If there are restrictions on sharing of data and/or materials, please state these. Please note that we cannot proceed with consideration of your article until this information has been declared.

Please include your amended Competing Interests Statement within your cover letter. We will change the online submission form on your behalf.

4. Please include your tables as part of your main manuscript and remove the individual files. Please note that supplementary tables (should remain/ be uploaded) as separate "supporting information" files"

5. If the reviewer comments include a recommendation to cite specific previously published works, please review and evaluate these publications to determine whether they are relevant and should be cited. There is no requirement to cite these works unless the editor has indicated otherwise.

[Note: HTML markup is below. Please do not edit.]

Reviewers' comments:

Reviewer's Responses to Questions

**Comments to the Author**

1. Is the manuscript technically sound, and do the data support the conclusions?

The manuscript must describe a technically sound piece of scientific research with data that supports the conclusions. Experiments must have been conducted rigorously, with appropriate controls, replication, and sample sizes. The conclusions must be drawn appropriately based on the data presented.

Reviewer #1: No

Reviewer #2: Yes

**********

2. Has the statistical analysis been performed appropriately and rigorously?

Reviewer #1: No

Reviewer #2: I Don't Know

**********

3. Have the authors made all data underlying the findings in their manuscript fully available?

The PLOS Data policy requires authors to make all data underlying the findings described in their manuscript fully available without restriction, with rare exception (please refer to the Data Availability Statement in the manuscript PDF file). The data should be provided as part of the manuscript or its supporting information, or deposited to a public repository. For example, in addition to summary statistics, the data points behind means, medians and variance measures should be available. If there are restrictions on publicly sharing data—e.g. participant privacy or use of data from a third party—those must be specified.

Reviewer #1: No

Reviewer #2: Yes

**********

4. Is the manuscript presented in an intelligible fashion and written in standard English?

PLOS ONE does not copyedit accepted manuscripts, so the language in submitted articles must be clear, correct, and unambiguous. Any typographical or grammatical errors should be corrected at revision, so please note any specific errors here.

Reviewer #1: Yes

Reviewer #2: Yes

**********

5. Review Comments to the Author

Please use the space provided to explain your answers to the questions above. You may also include additional comments for the author, including concerns about dual publication, research ethics, or publication ethics. (Please upload your review as an attachment if it exceeds 20,000 characters)

Reviewer #1: Dear Authors

Thank you for submitting your research for publication. This manuscript evaluates a formalin-free, supercritical CO₂–based tissue processing method (NFPE) versus FFPE, focusing on DNA/RNA integrity and RNA NGS performance. The topic is timely and relevant to molecular pathology, particularly given the increasing reliance on nucleic acid–based diagnostics and concerns regarding formalin-induced artifacts. However, there are substantial methodological and interpretational limitations that currently weaken the validity of the conclusions.

#Major Comments

1. Study Design and Cohort Heterogeneity

The study includes a relatively small number of samples with substantial heterogeneity in tissue types (e.g., colon, placenta, adrenal, myocardium, various tumors), without stratification or adjustment. Different tissue types have inherently DNA/RNA preservation characteristics. Additionally there was no stratification by tissue type, tumor vs normal status, or cellular composition.

Recommendation:Provide detailed breakdowns of sample numbers per tissue type and per group. Consider stratified analysis or justify pooling across heterogeneous tissues. Clarify whether matched comparisons are consistently applied.

2. Confounding Pre-analytical Variables

Key pre-analytical factors (Cold ischemia time, Time from excision to processing) are insufficiently controlled or described.

Recommendation:Clarify and standardize pre-analytical conditions where possible. Explicitly discuss this limitation and its potential impact on RNA and DNA integrity results.

3. Definition and Interpretation of False Positives

The classification of fusion calls in FFPE samples as “false positives” is not sufficiently supported by the data. In the Results section (lines 204–213), the authors report that several FFPE samples showed positive fusion calls, which were absent in matched NFPE samples and subsequently negative on an alternative assay. Based on this, the authors conclude that “indeed all positive results… were false-positive.” This interpretation is reiterated in the Discussion section (lines 273–278).

However, discordance between methods and negative results on a secondary assay do not definitively establish false positivity, particularly in the absence of an independent gold-standard validation (e.g., orthogonal sequencing, RT-PCR). Alternative explanations such as low-level true events, sampling variability/ tumor heterogeneity, or assay sensitivity differences are not excluded.

Recommendation: Define clear criteria for false-positive classification and provide supporting data. I suggest avoiding definitive claims without rigorous validation.

4. Statistical Analysis

The statistical approach is limited to t-tests, with no indication of:

* Adjustment for multiple comparisons

* Reporting of effect sizes or confidence intervals

Given the matched nature of samples and potential non-normal distributions, the current analysis may not be appropriate.

Recommendation:Reassess statistical methods. Consider non-parametric or paired analyses and report effect sizes alongside p-values.

5. Conflict of Interest and Potential Bias

The corresponding author is listed as an inventor on a patent related to the NFPE technology, and the study uses proprietary equipment (TISPA system). This may represent a significant potential conflict of interest.

Recommendation:Ensure that the role of the authors in developing the technology is transparently described. Consider including statements regarding measures taken to minimize bias. Independent validation would strengthen the manuscript.

6. Reproducibility and Generalizability

The study is conducted at a single center using a single platform (Ion Torrent), with no external validation.

Recommendation:Temper conclusions regarding broad applicability. Explicitly acknowledge limitations in reproducibility and generalizability.

7. Methodological Details and Reproducibility:

Important methodological details are missing or unclear:

* Input amounts for DNA/RNA extraction

* Extraction efficiency

* QC thresholds for NGS

*Library preparation success rates

* Potential batch effects

Recommendation:Expand the Methods section to ensure reproducibility and transparency where possible and expand on study limitations as required.

#Minor suggestions/comments

* The Discussion contains statements that are somewhat promotional. I recommend these toned down.

* Ensure consistent use of terms (e.g., NFPE vs “non-fixed”, FFPE vs “formalin-fixed”) in the manuscript.

* Figures:

Include sample sizes in figure legends.

Consider adding individual data points to boxplots.

* Data Availability

The statement that “all relevant data are within the manuscript and supporting files” is insufficient for a study involving NGS data.

Recommendation:Deposit sequencing data in a public repository (e.g., GEO, SRA) and provide accession numbers.

Kind regards

Reviewer #2: Abstract

• The phrase “falsely encourage personalized therapy” is overly strong/speculative for an abstract unless directly demonstrated by clinical validation. I recommend revising to more cautious wording.

Introduction

• Since the authors cite their own previous work demonstrating histologic and preliminary DNA quality benefits, the introduction should more clearly define how the current study advances beyond prior publications. The authors need to distinguish what was previously known, what remains unknown, and what new contribution this study provides.

Material and methods

• The authors need to specify the following: total number of tissue specimens included, number of samples per tissue type, whether samples were paired/matched between FFPE and NFPE conditions, and whether the same tissue specimen was split for comparative processing.

• Please justify the rationale for combining these diverse tissue types in the same analysis and discuss whether tissue-specific differences in cellularity/composition may influence DNA/RNA integrity outcomes.

• The authors used two different processors (TISPA I vs. TISPA II), thus introducing a potential technical confounder. Please explain the following: what differences exist between TISPA I and TISPA II systems, Whether protocol parameters differed between instruments, and why 2-year-old samples were processed differently than 3-day/7-year cohorts.

• The statement “stored under exactly the same conditions” is insufficiently specific. Please provide quantitative storage parameters: exact temperature range and whether storage was continuous and monitored.

• The authors need to specify the following: the total number of RNA samples subjected to NGS, how samples were selected from the larger cohort, and distribution of FFPE vs NFPE samples analyzed by NGS. This is essential for evaluating representativeness and study power.

Conclusion

• The sentence “RNA integrity in NFPE was lower than RNA integrity in young samples, but stable over time” is unclear and potentially confusing. It is not explicit whether “young samples” refers to young FFPE samples, young NFPE samples, or freshly processed tissues.

• The conclusion states NFPE is “quicker and cheaper” than FFPE. Unless cost/time analyses were formally performed in this study or cited robustly, these claims should be removed or qualified.

• Given that this is likely an early/technical validation study, the conclusion should acknowledge that: further multicenter validation, broader tissue-type testing, and workflow implementation studies may be needed before clinical adoption.

**********

6. PLOS authors have the option to publish the peer review history of their article (what does this mean?). If published, this will include your full peer review and any attached files.

If you choose “no”, your identity will remain anonymous but your review may still be made public.

**Do you want your identity to be public for this peer review?**  For information about this choice, including consent withdrawal, please see our Privacy Policy.

Reviewer #1: No

Reviewer #2: **Yes:**  Hoda Y Abdallah

**********

[NOTE: If reviewer comments were submitted as an attachment file, they will be attached to this email and accessible via the submission site. Please log into your account, locate the manuscript record, and check for the action link "View Attachments". If this link does not appear, there are no attachment files.]

To ensure your figures meet our technical requirements, please review our figure guidelines: https://journals.plos.org/plosone/s/figures

You may also use PLOS’s free figure tool, NAAS, to help you prepare publication quality figures: https://journals.plos.org/plosone/s/figures#loc-tools-for-figure-preparation.

NAAS will assess whether your figures meet our technical requirements by comparing each figure against our figure specifications.

# Author comment: Author response to Decision Letter 1

**DOI:** 10.1371/journal.pone.0356001.r002

9 Jun 2026

Journal Requirements:

When submitting your revision, we need you to address these additional requirements.

1. Please ensure that your manuscript meets PLOS ONE's style requirements, including those for file naming. The PLOS ONE style templates can be found at

https://journals.plos.org/plosone/s/file?id=wjVg/PLOSOne_formatting_sample_main_body.pdf and

https://journals.plos.org/plosone/s/file?id=ba62/PLOSOne_formatting_sample_title_authors_affiliations.pdf

2. We note that the grant information you provided in the ‘Funding Information’ and ‘Financial Disclosure’ sections do not match.

-In this new submission, we have checked the data and information and strive to be fair and complete.

When you resubmit, please ensure that you provide the correct grant numbers for the awards you received for your study in the ‘Funding Information’ section.

3. Thank you for stating the following in the Financial Disclosure section:

“Initials of the authors who received each award: AMH

Grant numbers awarded to each author: 1

The full name of each funder: Novartis Nederland

URL of each funder website: https://www.novartis.com/nl-nl/

Did the sponsors or funders play any role in the study design, data collection and analysis, decision to publish, or preparation of the manuscript? No, the funders had no role in study design, data collection and analysis, decision to publish, or preparation of the manuscript.”

-We note that you received funding from a commercial source: Novartis Nederland

Please provide an amended Competing Interests Statement that explicitly states this commercial funder, along with any other relevant declarations relating to employment, consultancy, patents, products in development, marketed products, etc.

Within this Competing Interests Statement, please confirm that this does not alter your adherence to all PLOS ONE policies on sharing data and materials by including the following statement:

We can confirm "This does not alter our adherence to PLOS ONE policies on sharing data and materials.” This extra sentence of text is added in the financial disclosure section. (as detailed online in our guide for authors http://journals.plos.org/plosone/s/competing-interests). If there are restrictions on sharing of data and/or materials, please state these. Please note that we cannot proceed with consideration of your article until this information has been declared.

Please include your amended Competing Interests Statement within your cover letter. We will change the online submission form on your behalf.

-We have included an amended statement.

4. Please include your tables as part of your main manuscript and remove the individual files. Please note that supplementary tables (should remain/ be uploaded) as separate "supporting information" files".

-We have now included the table as part of the main manuscript in the revised versions.

5. If the reviewer comments include a recommendation to cite specific previously published works, please review and evaluate these publications to determine whether they are relevant and should be cited. There is no requirement to cite these works unless the editor has indicated otherwise.

-The reviewers did not recommend any literature.

[Note: HTML markup is below. Please do not edit.]

Reviewers' comments:

Reviewer's Responses to Questions

Comments to the Author

1. Is the manuscript technically sound, and do the data support the conclusions?

The manuscript must describe a technically sound piece of scientific research with data that supports the conclusions. Experiments must have been conducted rigorously, with appropriate controls, replication, and sample sizes. The conclusions must be drawn appropriately based on the data presented.

Reviewer #1: No

Reviewer #2: Yes

________________________________________

2. Has the statistical analysis been performed appropriately and rigorously?

Reviewer #1: No

Reviewer #2: I Don't Know

________________________________________

3. Have the authors made all data underlying the findings in their manuscript fully available?

The PLOS Data policy requires authors to make all data underlying the findings described in their manuscript fully available without restriction, with rare exception (please refer to the Data Availability Statement in the manuscript PDF file). The data should be provided as part of the manuscript or its supporting information, or deposited to a public repository. For example, in addition to summary statistics, the data points behind means, medians and variance measures should be available. If there are restrictions on publicly sharing data—e.g. participant privacy or use of data from a third party—those must be specified.

Reviewer #1: No

Reviewer #2: Yes

________________________________________

4. Is the manuscript presented in an intelligible fashion and written in standard English?

PLOS ONE does not copyedit accepted manuscripts, so the language in submitted articles must be clear, correct, and unambiguous. Any typographical or grammatical errors should be corrected at revision, so please note any specific errors here.

Reviewer #1: Yes

Reviewer #2: Yes

________________________________________

5. Review Comments to the Author

Please use the space provided to explain your answers to the questions above. You may also include additional comments for the author, including concerns about dual publication, research ethics, or publication ethics. (Please upload your review as an attachment if it exceeds 20,000 characters)

Reviewer #1: Dear Authors

Thank you for submitting your research for publication. This manuscript evaluates a formalin-free, supercritical CO₂–based tissue processing method (NFPE) versus FFPE, focusing on DNA/RNA integrity and RNA NGS performance. The topic is timely and relevant to molecular pathology, particularly given the increasing reliance on nucleic acid–based diagnostics and concerns regarding formalin-induced artifacts. However, there are substantial methodological and interpretational limitations that currently weaken the validity of the conclusions.

#Major Comments

1. Study Design and Cohort Heterogeneity

The study includes a relatively small number of samples with substantial heterogeneity in tissue types (e.g., colon, placenta, adrenal, myocardium, various tumors), without stratification or adjustment. Different tissue types have inherently DNA/RNA preservation characteristics. Additionally there was no stratification by tissue type, tumor vs normal status, or cellular composition.

Recommendation:Provide detailed breakdowns of sample numbers per tissue type and per group. Consider stratified analysis or justify pooling across heterogeneous tissues.

We now explain that we omitted certain tissue types based on literature and included 2 new references.

Clarify whether matched comparisons are consistently applied.

-We now explain in the materials and methods that we pooled samples, omitted unsuitable samples and we included a new supplementary file showing matched comparisons of individual tissues that were split and processed as NFPE and FFPE.

2. Confounding Pre-analytical Variables

Key pre-analytical factors (Cold ischemia time, Time from excision to processing) are insufficiently controlled or described.

Recommendation: Clarify and standardize pre-analytical conditions where possible. Explicitly discuss this limitation and its potential impact on RNA and DNA integrity results.

-We included a new supplementary file and included preanalytical factors, we discus the limitations in the text of the revised manuscript.

3. Definition and Interpretation of False Positives

The classification of fusion calls in FFPE samples as “false positives” is not sufficiently supported by the data. In the Results section (lines 204–213), the authors report that several FFPE samples showed positive fusion calls, which were absent in matched NFPE samples and subsequently negative on an alternative assay. Based on this, the authors conclude that “indeed all positive results… were false-positive.” This interpretation is reiterated in the Discussion section (lines 273–278).

However, discordance between methods and negative results on a secondary assay do not definitively establish false positivity, particularly in the absence of an independent gold-standard validation (e.g., orthogonal sequencing, RT-PCR). Alternative explanations such as low-level true events, sampling variability/ tumor heterogeneity, or assay sensitivity differences are not excluded.

Recommendation: Define clear criteria for false-positive classification and provide supporting data. I suggest avoiding definitive claims without rigorous validation.

-To assess the validity of these fusion calls, we used the Archer FusionPlex assay. In this assay, the anchored multiplex PCR (AMP) chemistry is highly sensitive and specific; it can detect low-abundance transcripts. We now explain this more clearly in the revised manuscript and explain that the likelyhood of missing true-positive fusions by using this assay is very low (included additional references). In addition, we also downgraded the interpretation and omitted definitive claims about false positivity as suggested by the reviewer.

4. Statistical Analysis

The statistical approach is limited to t-tests, with no indication of:

* Adjustment for multiple comparisons

* Reporting of effect sizes or confidence intervals

Given the matched nature of samples and potential non-normal distributions, the current analysis may not be appropriate.

Recommendation: Reassess statistical methods. Consider non-parametric or paired analyses and report effect sizes alongside p-values.

-We now explain more clearly that we pooled the data and why we omitted certain tissue types. In addition we included a new supplementary file showing RIN and DIN values in matched comparisons of individual tissues that were split and processed as NFPE and FFPE.

5. Conflict of Interest and Potential Bias

The corresponding author is listed as an inventor on a patent related to the NFPE technology, and the study uses proprietary equipment (TISPA system). This may represent a significant potential conflict of interest.

Recommendation:Ensure that the role of the authors in developing the technology is transparently described. Consider including statements regarding measures taken to minimize bias. Independent validation would strengthen the manuscript.

-Primary interpretation of molecular data was done by the first author MN, a clinical molecular biologist. AMH, a pathologist, is only included in the patent to academically link the data to the Isala hospital and has no personal financial interest. The general statement will be included as requested by the editor.

6. Reproducibility and Generalizability

The study is conducted at a single center using a single platform (Ion Torrent), with no external validation.

Recommendation: Temper conclusions regarding broad applicability. Explicitly acknowledge limitations in reproducibility and generalizability.

-We tempered the conclusions in the revised manuscript and, in addition to the statement already made in the discussion, we also included a statement acknowledging limitations in reproducibility and generalizability (as also suggested by reviewer 2) in the conclusion.

7. Methodological Details and Reproducibility:

Important methodological details are missing or unclear:

*Input amounts for DNA/RNA extraction We now show this in supplementary figure 1

*Extraction efficiency

-We now show DNA and RNA concentrations in supplementary figure 1

*QC thresholds for NGS We now mention the QC thresholds for NGS in the methods section.

-We added the QC thresholds to materials and methods

*Library preparation success rates.

-Library preparation success rates (and failures) are shown in table 1.

*Potential batch effects.

-We did not notice batch effects.

Recommendation:Expand the Methods section to ensure reproducibility and transparency where possible and expand on study limitations as required.

-We have expanded the methods section, and we added a supplementary figure that includes additional information requested by the reviewers.

#Minor suggestions/comments

* The Discussion contains statements that are somewhat promotional. I recommend these toned down.

-We have toned down promotional statements throughout the revised manuscript.

* Ensure consistent use of terms (e.g., NFPE vs “non-fixed”, FFPE vs “formalin-fixed”) in the manuscript.

-We made adjustments to the revised manuscript to make this clearer.

* Figures:

Include sample sizes in figure legends.

-We now include samples size in the figure legends and show individual results in supplementary figure 1

Consider adding individual data points to boxplots.

-We now show individual datapoints in supplementary figure 1

* Data Availability

The statement that “all relevant data are within the manuscript and supporting files” is insufficient for a study involving NGS data.

Recommendation: Deposit sequencing data in a public repository (e.g., GEO, SRA) and provide accession numbers.

-We used Thermofisher sequencing equipment, which unfortunately generates BAM files instead of the required files (e.g. FASTQ, SRA) required for submission to GEO or SRA (or similar). The BAM files are available on request; we now added this statement to the manuscript.

# Editor report: Decision Letter 1

**DOI:** 10.1371/journal.pone.0356001.r003

**Authors:** Nasar Alwahaibi

28 Jul 2026

Good DNA- and sufficient RNA integrity in formalin-free paraffin-embedded tissues

PONE-D-26-13956R1

Dear Dr. Hoogland,

We’re pleased to inform you that your manuscript has been judged scientifically suitable for publication and will be formally accepted for publication once it meets all outstanding technical requirements.

Within one week, you’ll receive an e-mail detailing the required amendments. When these have been addressed, you’ll receive a formal acceptance letter and your manuscript will be scheduled for publication.

An invoice will be generated when your article is formally accepted. Please note, if your institution has a publishing partnership with PLOS and your article meets the relevant criteria, all or part of your publication costs will be covered. Please make sure your user information is up-to-date by logging into Editorial Manager at Editorial Manager® and clicking the ‘Update My Information' link at the top of the page. For questions related to billing, please contact billing support.

If your institution or institutions have a press office, please notify them about your upcoming paper to help maximize its impact. If they’ll be preparing press materials, please inform our press team as soon as possible -- no later than 48 hours after receiving the formal acceptance. Your manuscript will remain under strict press embargo until 2 pm Eastern Time on the date of publication. For more information, please contact onepress@plos.org.

Kind regards,

Nasar Alwahaibi, PhD

Academic Editor

PLOS One

Additional Editor Comments (optional):

Reviewers' comments:

# Editor report: Acceptance letter

**DOI:** 10.1371/journal.pone.0356001.r004

**Authors:** Nasar Alwahaibi

PONE-D-26-13956R1

PLOS One

Dear Dr. Hoogland,

I'm pleased to inform you that your manuscript has been deemed suitable for publication in PLOS One. Congratulations! Your manuscript is now being handed over to our production team.

At this stage, our production department will prepare your paper for publication. This includes ensuring the following:

* All references, tables, and figures are properly cited

* All relevant supporting information is included in the manuscript submission,

* There are no issues that prevent the paper from being properly typeset

You will receive further instructions from the production team, including instructions on how to review your proof when it is ready. Please keep in mind that we are working through a large volume of accepted articles, so please give us a few days to review your paper and let you know the next and final steps.

Lastly, if your institution or institutions have a press office, please let them know about your upcoming paper now to help maximize its impact. If they'll be preparing press materials, please inform our press team within the next 48 hours. Your manuscript will remain under strict press embargo until 2 pm Eastern Time on the date of publication. For more information, please contact onepress@plos.org.

You will receive an invoice from PLOS for your publication fee after your manuscript has reached the completed accept phase. If you receive an email requesting payment before acceptance or for any other service, this may be a phishing scheme. Learn how to identify phishing emails and protect your accounts at https://explore.plos.org/phishing.

If we can help with anything else, please email us at customercare@plos.org.

Thank you for submitting your work to PLOS ONE and supporting open access.

Kind regards,

PLOS ONE Editorial Office Staff

on behalf of

Dr. Nasar Alwahaibi

Academic Editor

PLOS One
